# Towards photon radiotherapy treatment planning with high Z nanoparticle radiosensitisation agents: the Relative Biological Effective Dose (RBED) framework

**DOI:** 10.1186/s12645-018-0043-7

**Published:** 2018-11-09

**Authors:** Jeremy M. C. Brown, Gerard G. Hanna, Nathanael Lampe, Balder Villagomez-Bernabe, James R. Nicol, Jonathan A. Coulter, Fred J. Currell

**Affiliations:** 1 School of Mathematics and Physics, Queen’s University Belfast, Belfast, Northern Ireland UK; 2 Department of Radiation Science and Technology, Delft University of Technology, Delft, The Netherlands; 3 Centre for Medical Radiation Physics, University of Wollongong, Wollongong, Australia; 4 School of Medicine, Dentistry and Biomedical Sciences, Queen’s University Belfast, Belfast, Northern Ireland UK; 5 Université Clermont Auvergne, CNRS/IN2P3, LPC, Clermont-Ferrand, France; 6 School of Pharmacy, Queen’s University Belfast, Belfast, Northern Ireland UK

**Keywords:** Radiotherapy, Nanoparticles, Treatment planning, Radiosensitisers, Biological effect modelling, Theranostic nanoparticles

## Abstract

A novel treatment planning framework, the Relative Biological Effective Dose (RBED), for high Z nanoparticle (NP)-enhanced photon radiotherapy is developed and tested in silico for the medical exemplar of neoadjuvant (preoperative) breast cancer MV photon radiotherapy. Two different treatment scenarios, conventional and high Z NP enhanced, were explored with a custom Geant4 application that was developed to emulate the administration of a single 2 Gy fraction as part of a 50 Gy radiotherapy treatment plan. It was illustrated that there was less than a 1% difference in the dose deposition throughout the standard and high Z NP-doped adult female phantom. Application of the RBED framework found that the extent of possible biological response with high Z NP doping was great than expected via the dose deposition alone. It is anticipated that this framework will assist the scientific community in future high Z NP-enhanced in-silico, pre-clinical and clinical trials.

## Introduction

Photon radiotherapy is one of the most commonly employed approaches in the treatment of cancer (Delaney et al. 2005; Urruticoechea et al. 2010). Since its first application in the 1920s, photon radiotherapy has undergone continuous refinement through the development of new technologies and increased understanding of radiation biology (Mayles et al. 2007; Joiner and van der Kogel 2009). Further optimisation has included the combination of radiotherapy and systemic therapy with resultant improvements in both local control and overall survival (Aupérin et al. 2010). Over the last decade, there has been increasing interest in the use of nanomedicines both as single-agent anti-cancer therapeutics and in combination with radiotherapy (Chen et al. 2016; Schuemann et al. 2016; Marples and Dhar 2017). A novel class of nanomedicines, high Z nanoparticle (NP) radiosensitisation agents, that possess the potential to further increase the efficacy of photon radiotherapy have recently entered the first phase of clinical trials (National Institutes of Health (USA) 2016). These nanomedicines can be functionalised to seek out cancerous cells/tumours and when irradiated increase the local energy deposition and free-radical yield within a few tens to hundreds of nanometres surrounding each NP (Hainfeld et al. 2004, 2008; Jones et al. 2010; McMahon et al. 2011; Jain et al. 2012; Lechtman et al. 2013; Lin et al. 2014; Sicard-Roselli et al. 2014; Tran et al. 2016). However, the majority of in-vitro studies exploring the use of these nanomedicines for photon radiotherapy applications illustrate that the biological response of high Z NP-doped cells do not directly scale with these factors alone (McMahon et al. 2011; Lechtman et al. 2013; Chithrani et al. 2010; Liu et al. 2010; Jain et al. 2011; Sancey et al. 2014; Nicol 2016).

The complex nature of biological response of high Z NP-doped cells under irradiation from clinical photon radiotherapy sources presents a significant challenge when developing accurate treatment planning schemes. Initially, investigators attempted to apply a variety of Dose Enhancement Figures of Merit (DEFM) to evaluate the possible potential of high Z NP enhancement photon radiotherapy in the clinical setting (Cho 2005; Roeske et al. 2007; Ngwa et al. 2010). These DEFMs were based on the assumption that the expected biological outcome of cells/tumours could be described via the ratio of dose deposition with and without high Z NP doping under uniform photon irradiation, a concept now known to possess limited validity (McMahon et al. 2011; Lechtman et al. 2013; Chithrani et al. 2010; Liu et al. 2010; Jain et al. 2011; Sancey et al. 2014). For a high Z NP-enhanced photon radiotherapy treatment planning framework to be applicable to the clinical setting, it would need to account for the complex biological behaviour of NP-doped cells whilst conveying the improved outcome in terms comparable with conventional photon radiotherapy.

Recently Ferrero et al. (2017) and Strigari et al. (2017) presented a two article series on the treatment planning of radiotherapy for breast cancer using gold nanoparticles (AuNP). The first of these papers focused on the reformulation of the local effect model (LEM) to estimate AuNP-driven increase in radiosensitivity. Here a physics-driven additive approach was implemented based on the average number of ionisations per AuNP per Gy of dose, and the average number of AuNPs located inside or adjacent to the cell nucleus (Ferrero et al. 2017). In the second paper, this formalism was applied to the treatment planning of breast cancer radiotherapy with 2-nm-diameter AuNPs utilising Eclipse v.8.9 (Varian Medical Systems, Palo Alto, CA, USA) (Strigari et al. 2017). From these works, it was illustrated that an improved treatment efficacy outcome is possible based on the developed formalism and selected targeting proprieties of the AuNPs. However, as highlighted in Ferrero et al. this physics-driven approach neglects two major sets of physiological factors that underpin the biological response of NP-doped cells under irradiation: variation and localisation of AuNP uptake within/surrounding cells, and impact of AuNPs on toxicity and cellular cycle/repair processes (Ferrero et al. 2017).

This work proposes a biological data-driven framework, the Relative Biological Effective Dose (RBED), that aims to enable pre-clinical and clinical treatment planning of high Z NP-enhanced photon radiotherapy. It combines the Relative Biological Effectiveness (RBE) phenomenological model for proton radiotherapy outlined in Wilkens and Oelfke (2004), with the experimentally benchmarked NP radiosensitisation interpolation framework of Brown and Currell (2017) to convey the observed increased biological effect in terms of conventional photon radiotherapy dose. The framework of Brown and Currell was developed to leverage available photon-NP biological radiosensitisation data to predict biological response with the specific purpose of accounting for the physiological effects associated with variation and localisation of AuNP uptake within/surrounding cells, and impact of NPs on toxicity and cellular cycle/repair processes. To illustrate the viability of the RBED framework as a pre-clinical/clinical treatment planning tool, an in-silico study of neoadjuvant (preoperative) high Z NP-enhanced breast cancer MV photon radiotherapy was undertaken utilising the Monte Carlo radiation transport modelling toolkit Geant4 (Agostinelli 2003; Allison 2006, 2016).

## Method and materials

### Relative Biological Effective Dose (RBED) framework

The RBED framework was developed to leverage available photon-NP biological radiosensitisation data to convey the increased biological response of high Z NP-enhanced photon radiotherapy on equal terms with conventional photon radiotherapy. In this work, two biological systems composed of the same cell line/tissue type are considered, one doped with a known concentration of a high Z NP radiosensitising agent (System A) and one without (System B). The survival fraction of these two systems after irradiation, $\mathrm {SF}_{\text{A}}$ for System A and $\mathrm {SF}_{\text{B}}$ for System B, can be described:

1 $$\begin{aligned} \mathrm {SF}_{\text{A}}= & {} \exp \left( -\alpha _{\text{A}}D_{\text{A}} - \beta _{\text{A}}D_{\text{A}}^2\right) , \end{aligned}$$

2 $$\begin{aligned} \mathrm {SF}_{\text{B}}= & {} \exp \left( -\alpha _{\text{B}}D_{\text{B}} - \beta _{\text{B}}D_{\text{B}}^2\right), \end{aligned}$$

where $D_{\text{A}}$ and $D_{\text{B}}$ are the administered radiation doses, and $\alpha _{\text{A}}$, $\alpha _{\text{B}}$, $\beta _{\text{A}}$ and $\beta _{\text{B}}$ are the fitted Linear Quadratic (LQ) model (Douglas and Fowler 1976) parameters of each respective system. These systems can be said to have biological equivalence when the survival fraction of their irradiated cell populations are equal:

3 $$\begin{aligned} \exp \left( -\alpha _{\text{A}}D_{\text{A}} - \beta _{\text{A}}D_{\text{A}}^2\right) = \exp \left( -\alpha _{\text{B}}D_{\text{B}} - \beta _{\text{B}}D_{\text{B}}^2\right) \end{aligned}$$

which, after further manipulation, yields

4 $$\begin{aligned} \displaystyle \beta _{\text{B}}D_{\text{B}}^2+\alpha _{\text{B}}D_{\text{B}} - (\alpha _{\text{A}} D_{\text{A}} + \beta _{\text{A}} D_{\text{A}}^2) = 0. \end{aligned}$$

Therefore, the RBED of the system containing the high Z NPs can be given via the positive solution for $D_{\text{B}}$ of this quadratic expression:

5 $$\begin{aligned} \displaystyle D_{\text{B}} = \frac{\sqrt{\alpha _{\text{B}}^{2}+4\beta _{\text{B}}\left( \alpha _{\text{A}}D_{\text{A}}+\beta _{\text{A}}D^{2}_{\text{A}}\right) }-\alpha _{\text{B}}}{2\beta _{\text{B}}} \end{aligned}$$

which is equivalent to Equation 3 in Wilkens and Oelfke (2004).

The key factor that dictates the accuracy of the phenomenological approach outlined above are the sourced values of each systems $\alpha$ and $\beta$. Whilst it is possible to obtain these values from a number different of sources, e.g. clonogenic cell assays, clinical trials, computational radiobiology, the majority of these high Z NP radiosensitisation studies for given cell line/tissue type are typically limited to two measurements for each tested photon source: a control and a single doped concentration $(C_{\text{M}})$. To enable the application of these datasets to a clinically relevant scenario, i.e. varied high Z NP concentrations (*C*) in different tissue regions, interpolated values for $\alpha _{\text{A}}$ and $\beta _{\text{A}}$ can be determined using the interpolation framework of Brown and Currell (2017), i.e.,

6 $$\begin{aligned} \displaystyle \alpha _{\text{A}}D_{\text{A}}+\beta _{\text{A}}D^{2}_{\text{A}} = \left( \alpha _{\text{B}}+\frac{C}{C_{\text{M}}}\Delta \alpha \right) D_{\text{A}} + \left( \beta _{\text{B}}+\frac{C}{C_{\text{M}}}\Delta \beta \right) D_{\text{A}}^{2} \end{aligned},$$

where $\Delta \alpha = \alpha _{\text{B}}(C_{\text{M}}) - \alpha _{\text{A}}$ and $\Delta \beta = \beta _{\text{B}}(C_{\text{M}}) - \beta _{\text{A}}$. Substitution of Eq. 6 into Eq. 5 gives the final form of the RBED framework:

7 $$\begin{aligned} \displaystyle D_{\text{B}} = \frac{\sqrt{\alpha _{\text{B}}^{2}+4\beta _{\text{B}}\left( \left( \alpha _{\text{B}}+\frac{C}{C_{\text{M}}}\Delta \alpha \right) D_{\text{A}}+ \left( \beta _{\text{B}}+\frac{C}{C_{\text{M}}}\Delta \beta \right) D_{\text{A}}^{2}\right) }-\alpha _{\text{B}}}{2\beta _{\text{B}}}. \end{aligned}$$

### In-silico study of high Z NP-enhanced breast cancer MV photon radiotherapy

An in-silico platform was developed to illustrate the potential of the RBED framework for the medical exemplar of neoadjuvant (preoperative) breast cancer MV photon radiotherapy (Calitchi et al. 2001; Riet et al. 2017) using the Monte Carlo radiation transport modelling toolkit Geant4 version 10.02.p02. The implemented simulation platform was designed to emulate the administration of a single 2 Gy fraction as part of a 50 Gy radiotherapy treatment plan across a two-compartment spherical tumour located in the left breast of an adult female. Two different treatment scenarios, conventional and high Z NP enhanced, were explored for a set of 1 Gy on-axis tumour-shaped projections with a 6 MV Varian Linac photon source. For the high Z NP-enhanced treatment scenario, it was assumed that a sufficient quantity of the popular commercial proprietary thiol-capped high Z NP radiosensitisation agent Aurovist (Hainfeld et al. 2004; Jain et al. 2011, 2012; Rahman et al. 2009; Bobyk et al. 2013; Al Zaki et al. 2014; Her et al. 2017), 1.9-nm-diameter gold nanoparticles (AuNPs), was administered to the patient to yield a maximum concentration within the two-compartment tumour of 500 μg/ml.

**Fig. 1:**
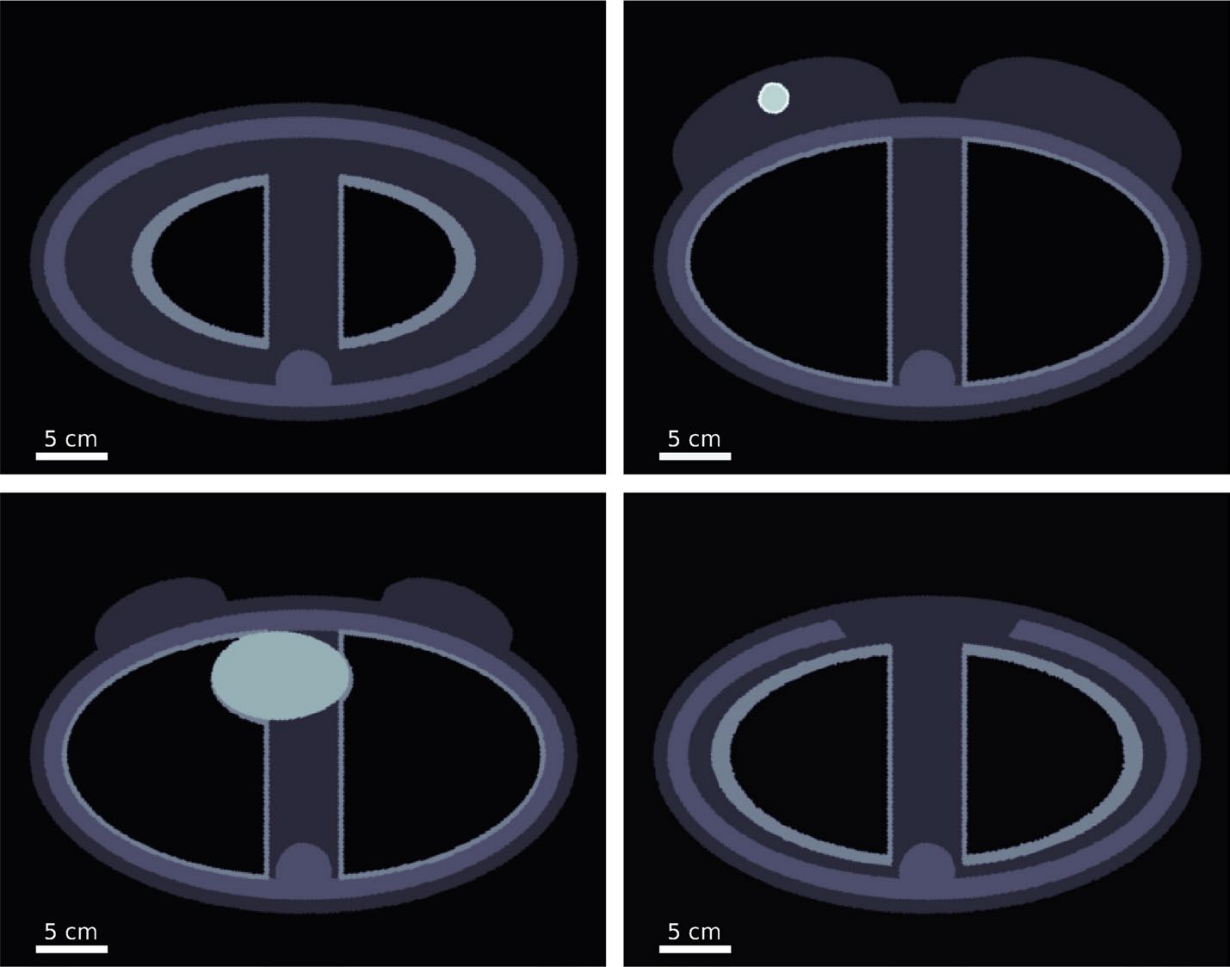
Cross-sectional view of the simulated adult female phantom at the centre of the first (top left), third (top right), fourth (bottom left) and sixth ribs (bottom right). In the top left and bottom right images, top and bottom cross sections of the lung volume can be seen, respectively. The top right cross section displays the central axis of the breasts and the two-compartment tumour in addition to the lung walls, whereas the bottom left cross section displays the central axis of the solid skewed ellipsoid representing the heart in addition to the lung walls. A voxelised version of the phantom can be obtained at the Delft University of Technology Research Data repository (http://researchdata.4tu.nl)

Figure 1 presents a set of cross-sectional views of the simulated adult female phantom. The simulated adult female phantom was composed of a soft tissue elliptical cylinder containing the C1 to C7 vertebra of the spine, sternum, the seven attached ribs, both lungs and solid skewed ellipsoid representing the heart. Two fused cylinder-ellipsoids representing the breasts were contoured onto the surface of the elliptical cylinder centred in front of the third rib. Each breast is rotated 20° way from the sternum to mimic their resting position for a patient lying on their back. Within the left breast, a two-compartment tumour of diameters 20 mm and 16 mm is located 20 mm in front of the breast centre away from the torso. In the case of the high Z NP-enhanced simulated irradiation, a uniform AuNP concentration of 250 μg/ml and 500 μg/ml was implemented for the inner and tumour wall regions, respectively, to approximate the uptake effects observed within solid tumours (Brown and Giaccia 1998; Minchinton and Tannock 2006; Hainfeld et al. 2011), whereas the remaining soft tissue and organs was set to 25 μg/ml to emulate the small animal uptake distributions for the Aurovist radiosensitisation agents observed in Hainfeld et al. (2006, 2011). A summary of the material types of the different phantom components and their relative AuNP concentrations is outlined in Table 1.

**Table 1:** Material type and ratio of AuNP uptake of each phantom component seen in Fig. 1

Phantom component	Material	AuNP uptake (μg/ml)
Ribs	ICRU-49 Compact Bone International Commission on Radiation Units and Measurements (1994)	0
Spine	ICRU-49 Compact Bone International Commission on Radiation Units and Measurements (1994)	0
Sternum	ICRU-49 Compact Bone International Commission on Radiation Units and Measurements (1994)	0
Heart	ICRU-44 Muscle International Commission on Radiation Units and Measurements (1989)	25
Lung wall	ICRU-44 Lung Tissue International Commission on Radiation Units and Measurements (1989)	25
Torso bulk	ICRU-44 Soft Tissue International Commission on Radiation Units and Measurements (1989)	25
Breast	ICRU-44 Breast Tissue International Commission on Radiation Units and Measurements (1989)	25
Tumour inner	ICRU-44 Breast Tissue International Commission on Radiation Units and Measurements (1989)	250
Tumour wall	ICRU-44 Breast Tissue International Commission on Radiation Units and Measurements (1989)	500

A voxelised version of the phantom can be obtained at the Delft University of Technology Research Data repository (http://researchdata.4tu.nl)

**Fig. 2:**
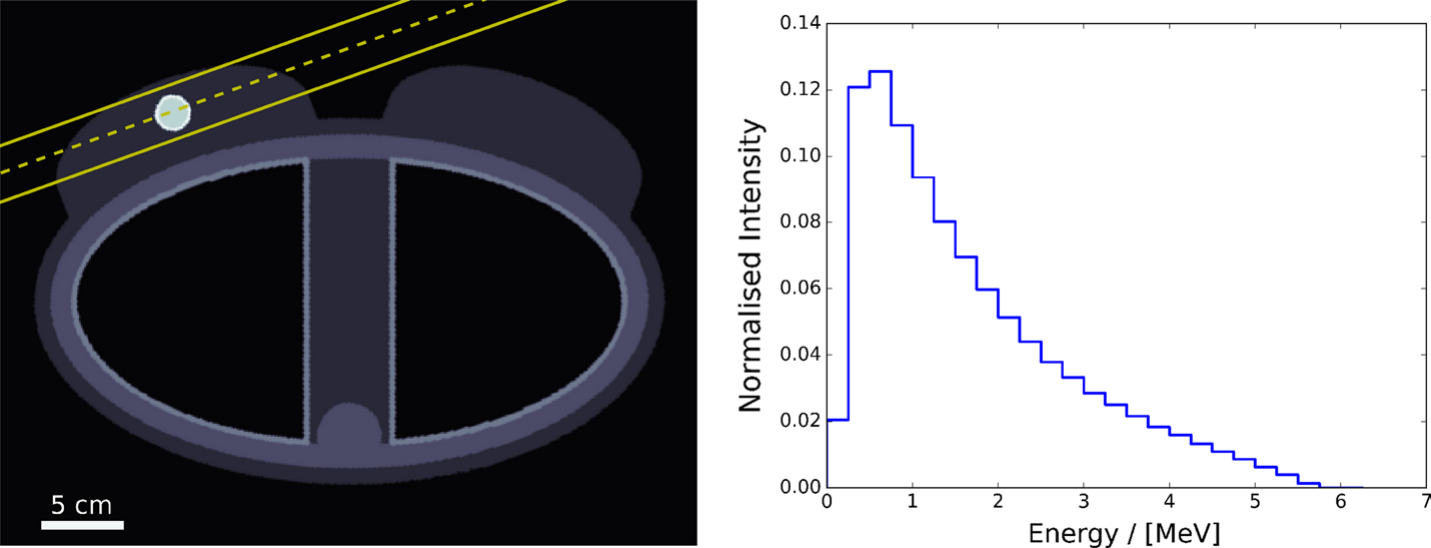
Cross-section of the simulated adult female phantom irradiation geometry (left) and primary photon energy spectra of the simulated 6 MV Varian Linac source taken from Sheikh-Bagheri and Rogers (2002) (right). The solid and dashed yellow lines represent the outer edges and central axis, respectively, of the 30-mm-diameter circular non-diverging photon beam rotated 20° off-axis focused at the two-compartment tumour centre located in the left breast

To achieve a minimum 2 Gy dose across the tumour and limit dose to non-tumour regions within the simulated phantom, the irradiation geometry, seen in Fig. 2 (left), was composed of a 30-mm-diameter circular non-diverging photon beam rotated 20° off-axis focused at the two-compartment tumour centre. Both treatment scenarios implemented two equal “exposure time” sub-fraction irradiations delivered in opposite directions along the dashed axis seen in Fig. 2 (left). Each sub-fraction irradiation simulated a total of $1.55\times 10^{12}$ primary photons of energy sampled from the 6 MV Varian Linac photon energy spectra presented in Fig. 2 (right). These simulations were undertaken at the macroscopic scale with 1 mm^3^ Dose Volume Histogram (DVH) voxelisation using the Geant4 Option4 EM physics configuration. Atomic deexcitation, a maximum particle step length of 250 μm and a low-energy particle cutoff of 1 keV, was implemented to simulate the transport of primary photons and their generated secondary particles throughout the phantom.

The radiosensitisation response parameters, i.e. $\alpha$ and $\beta$ values, for the AuNP-doped tissue types of the phantom were sourced from Jain et al. (2011). This study explored the radiosensitisation of three different human cell lines, DU145 prostate cancer cells, MDA-MB-231 breast cancer cells and L132 lung epithelial cells, for the selected AuNP agent and discovered that only the AuNP-doped MDA-MB-231 breast cancer cells saw increased sensitisation at megavoltage energies (6 MV and 15 MV). Based on these findings, and the fact that the expected AuNP uptake in health tissue is significantly lower than the tumour due to enhanced permeability and retention effects (Maeda 2012), it was assumed that only breast cells undergo an increased radiosensitisation with the selected AuNP agent at megavoltage photon energies. Therefore the heart, lung and soft tissue regions within the phantom were modelled as having a zero increase in radiosensitisation, whereas the breast tissue that makes up both breast and the tumour regions were modelled using the 6MV Varian Linac-irradiated MDA-MB-231 breast cancer cell parameters contained in Table 1 of Jain et al. (2011). To reflect the inherent uncertainty in-vitro clonogenic assays experiments, and the fundamental difference between cell culture and tissue response under irradiation, three different scenarios of radiobiological response was explored: minimum, mean and maximum relative AuNP agent radiosensitisation (see Table 2 for their $\alpha$ and $\beta$ parameter values).

**Table 2:** Phantom breast tissue $\alpha$ and $\beta$ values for the minimum, mean and maximum radiosensitisation response scenarios for high Z NP-enhanced photon radiotherapy with 1.9 nm Aurovist AuNPs under 6 MV Linac irradiation

Scenario	$\alpha$	$\beta$	$\alpha$	$\beta$
	(0 μg/ml)		(500 μg/ml)	
Minimum	0.024	0.086	0.064	0.087
Mean	0.002	0.079	0.104	0.098
Maximum	0.000	0.072	0.144	0.109

Data taken from Jain et al. (2011) MDA-MB-231 breast cancer cell response. Minimum case $\alpha$ and $\beta$ determine by minimising AuNP-doped and maximising conventional cell response within experimental uncertainty. Maximum case $\alpha$ and $\beta$ determine by maximising AuNP-doped and minimising conventional cell response within experimental uncertainty

## Results

### Dose deposition within the conventional and AuNP-doped phantom

**Fig. 3:**
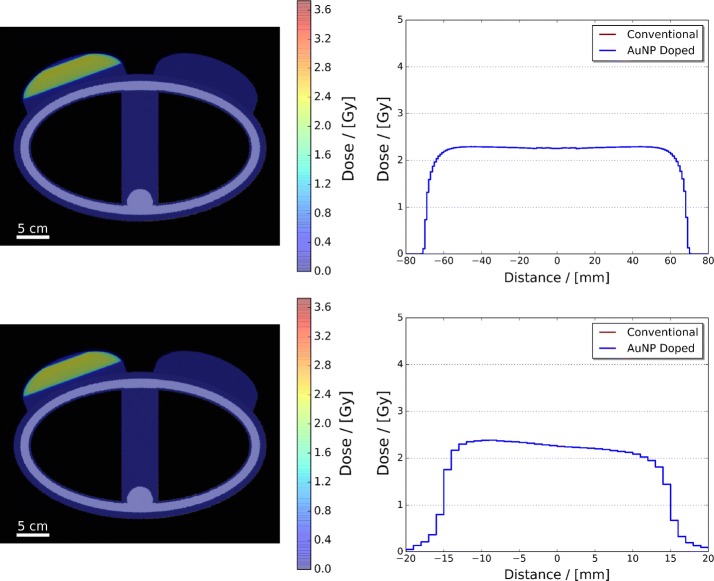
Dose maps of the adult female phantom, conventional (top left) and AuNP doped (bottom left), and accompanying parallel (top right) and perpendicular (bottom right) dose profiles with respect to the simulated central axis of the photon beam

**Fig. 4:**
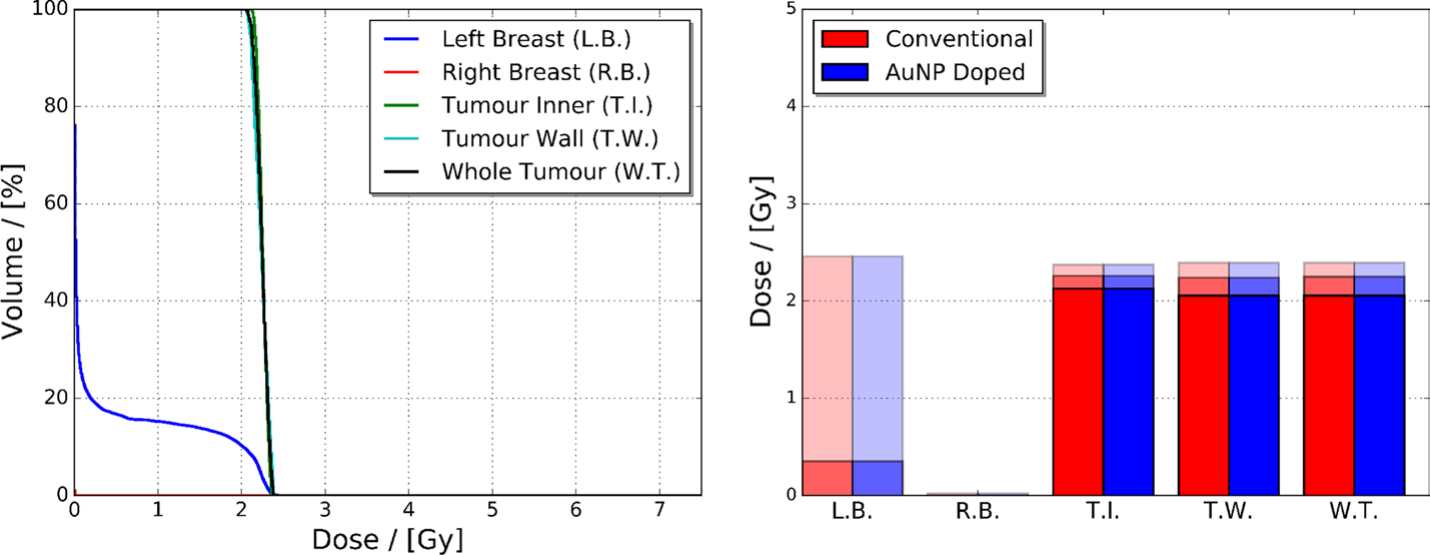
Dose Volume Histograms (DVHs) (left), and minimum, mean and maximum dose (right), illustrated via increasing transparency, of the simulated conventional and AuNP-doped adult female phantom breast tissue and whole tumour/tumour regions. The conventional (solid) and AuNP-doped (dashed) DVH profiles for the breast tissue and tumour regions differ by less than 1% causing an overlap

Figure 3 presents dose maps of the adult female phantom, both conventional and AuNP doped, and their parallel and perpendicular dose profiles with respect to the simulated central axis of the photon beam. Inspection of these dose maps and profiles illustrate that (1) in each treatment scenario the majority of the dose deposited within the phantom was limited to the target region defined via the edges of the shaped photon field outline in Fig. 2, and (2) the dose deposition in both the conventional and AuNP doped are effectively identical resulting in the overlap of their respective parallel and perpendicular profiles. The accompanying parallel dose profiles further illustrate that, as intended, the dose delivered throughout the left breast along the central axis of irradiation exceeded 2 Gy in both treatment scenarios, whereas the perpendicular dose profiles show the presence of a dose gradient across the central target region of the photon beam, ± 10 mm, from left to right that approaches 2 Gy at the tumour’s right edge. This gradient is the result of two primary physical factors: different extents of spectral hardening and intensity variation of the beam incident on the tumour surface [due to varied path lengths of beam through the left breast (Metcalfe et al. 1990)], and beam broadening due to photon scattering [illustrated via the Gaussian like roll-off after ± 15 mm (Ahnesjö and Aspradakis 1999)]. However, even with these factors the minimum dose administered to the whole tumour in both simulated treatment scenarios reached the desired target of 2 Gy (see Fig. 4).

Quantification of the observed minimal difference in the dose deposited between the two treatment scenarios can be visualised via each simulated phantom’s DVH, and minimum, mean and maximum breast tissue and whole tumour/tumour region delivered doses seen in Fig. 4. Comparison of each phantom regions DVHs and mean dose illustrates that there is less than 1% difference between the two treatment scenarios (which is on the same order of magnitude as the statistical accuracy of the simulation). Furthermore, both sets of DVHs illustrate that near-zero dose is deposited outside of the left breast and target tumour region.

### RBED within the conventional and AuNP-doped phantom

**Fig. 5:**
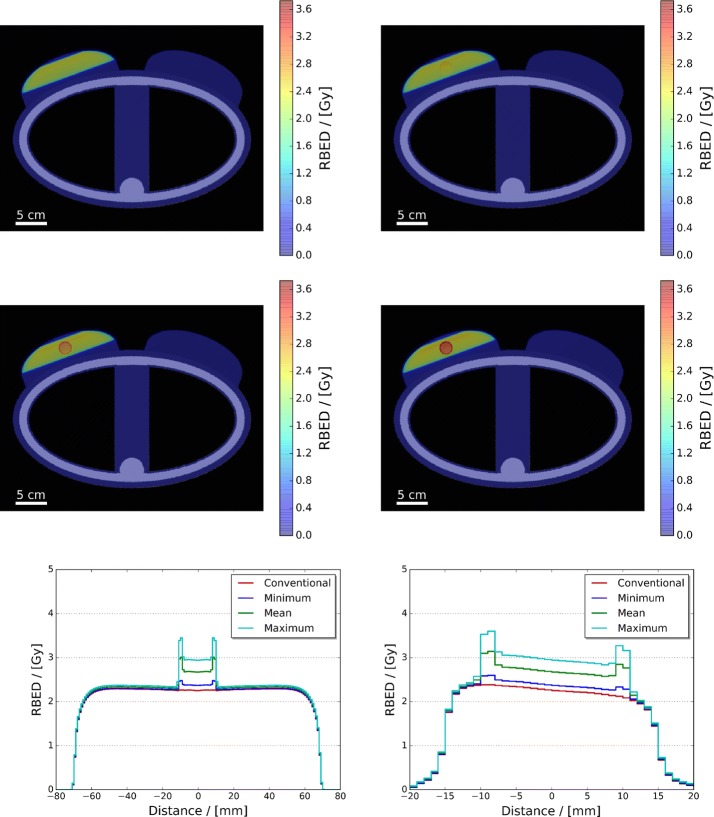
RBED maps of the irradiated adult female phantom for the conventional (top left), minimum (top right), mean (middle left) and maximum (middle right) relative AuNP agent radiosensitisation scenarios. Accompanying parallel and perpendicular RBED profiles with respect to the simulated central axis of the photon beam can be seen in the bottom left and right panels, respectively

Figure 5 presents the RBED maps of the irradiated adult female phantom for the conventional, minimum, mean and maximum relative AuNP agent radiosensitisation scenarios. With increasing relative radiosensitisation, the inner and tumour wall regions display an increased biological effect with respect to conventional radiotherapy (as indicated via increased RBED). The accompanying parallel and perpendicular RBED profiles of these maps further convey the extent of these increases within the inner and tumour wall regions of up to 3.08 and 3.67 Gy for the maximum radiosensitisation scenario, respectively. However, these RBED profiles also illustrate an increased biological effect in the left breast that scales proportionally with relative AuNP radiosensitisation. This observable increase within the left breast, which was less than 10% for all three relative radiosensitisation scenarios, highlights the need for correct selection of the high Z NP agent to ensure maximised uptake in the target irradiation volume whilst minimising uptake in the surrounding non-target tissue. Accounting for this effect is expected to be one of the most important considerations in high Z NP-enhanced photon radiotherapy treatment planning to ensure maximum tumour control probability whilst minimising normal tissue complications.

**Fig. 6:**
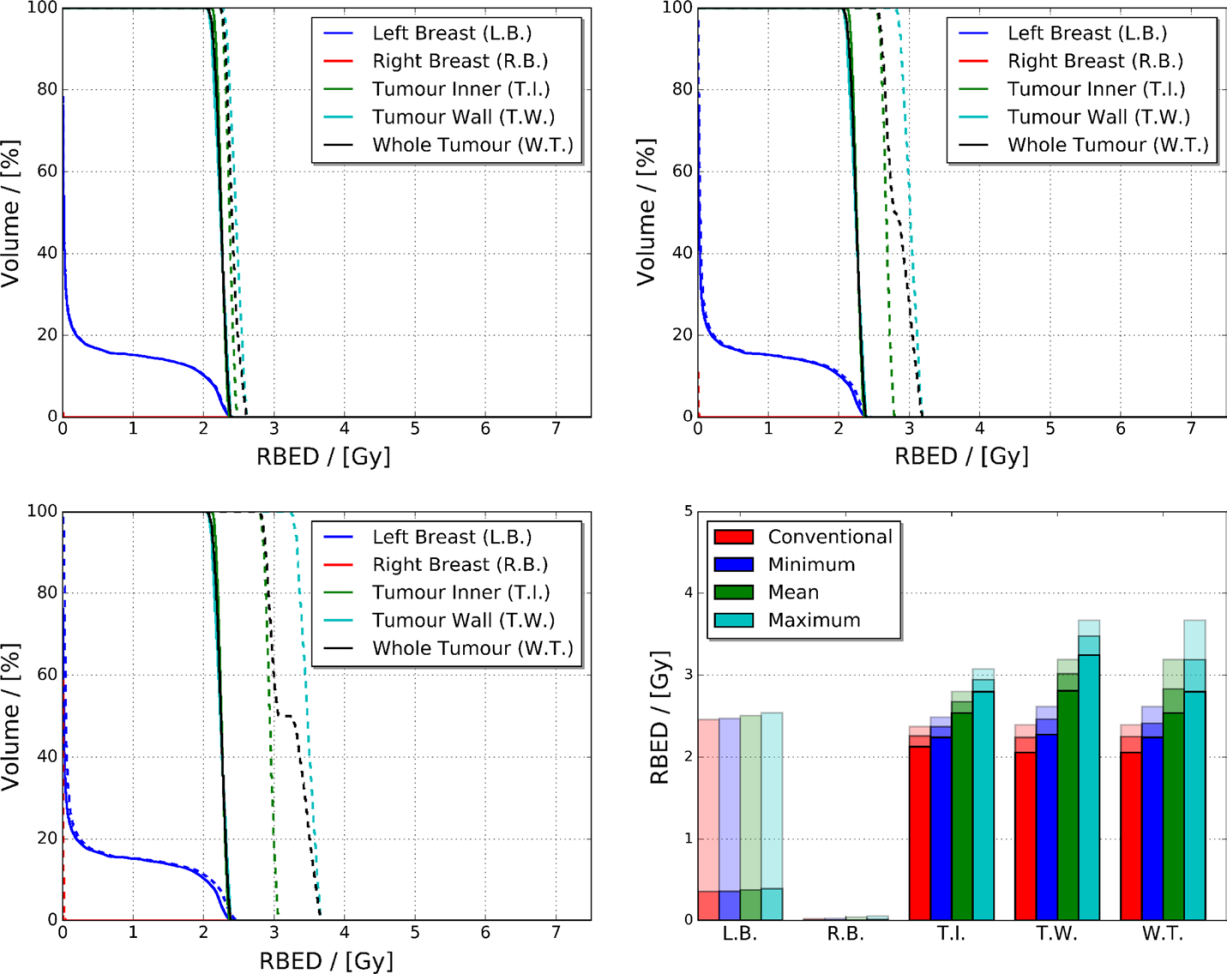
RBED Volume Histograms (RVHs) of the conventional and minimum (top left), mean (top right) and maximum (bottom left) relative AuNP agent radiosensitisation scenarios. Supporting minimum, mean and maximum RBED, illustrated via increasing transparency, of the simulated conventional, minimum, mean and maximum relative AuNP agent radiosensitisation adult female phantom breast tissue and whole tumour/tumour regions can be found in the bottom right panel

The RBED Volume Histograms (RVHs), and minimum, mean and maximum breast tissue and whole tumour/tumour region RBEDs for the conventional, minimum, mean and maximum relative AuNP agent radiosensitisation scenarios can be seen in Fig. 6. The observed trends in the RBED maps and profiles of Fig. 5 correlate with the increased RBED per unit volume of the left breast and tumour regions displayed in the RVHs. The extent of increase in mean RBED from the conventional to maximum relative AuNP agent radiosensitisation for the left and right breasts spans from 0.354 to 0.390 Gy and 0.002 to 0.012 Gy, respectively, whereas for the tumour inner, tumour wall and whole tumour the mean RBED increases from 2.258 to 2.945 Gy, 2.241 to 3.478 Gy, and 2.250 to 3.190 Gy.

## Discussion

A novel framework for pre-clinical and clinical treatment planning of high Z NP-enhanced photon radiotherapy was developed and its applicability tested in silico for the medical exemplar of neoadjuvant (preoperative) cancer MV photon radiotherapy. The simulations undertaken with the developed in-silico platform illustrated that for a semi-realistic uptake distribution of the selected AuNP radiosensitisation agent there was less than a 1% difference in the dose deposition throughout the standard and AuNP-doped adult female phantom. Application of the RBED framework for the three different relative AuNP agent radiosensitisation scenarios highlight that, even for this near-zero dose deposition difference, in every case an increase biological response was present in the left breast, right breast and tumour with respect to conventional radiotherapy. In fact, to achieve the same level of biological response within the tumour as the minimum, mean and maximum radiosensitisation scenarios, the conventional radiotherapy treatment approach would require a 9%, 33% and 53% increase in radiation exposure, respectively. Overall these results further illustrate the need of a framework such as RBED which is capable of accounting for the complex biological behaviour of NP-doped cells whilst conveying the improved outcome in terms comparable with conventional photon radiotherapy.

Whilst the findings of the present work point to the viability of the selected AuNP radiosensitisation agent for MV photon radiotherapy treatment of neoadjuvant breast cancer, due to a number of approximations it is difficult to draw a definite conclusion. Within the simulated adult female phantom, the distribution of the high Z NP radiosensitisation agent uptake was simplified to a generic geometry due to the lack of supporting patient data. In reality, it is expected that the distribution of the high Z NP agent uptake within the patent will differ throughout various tissue regions and the target tumour due to its complex vascular structure (Brown and Giaccia 1998; Minchinton and Tannock 2006). Moreover, the limited nature of available photon-NP biological radiosensitisation data forced a number of assumptions to be employed for the soft tissue response of the selected high Z NP agent under MV photon irradiation. If further supporting photon-NP biological radiosensitisation data and anatomical uptake distributions within realistic patient geometries could be obtained, it would be possible via the RBED framework to make an informed conclusion. However, the aim of this work was to introduce the RBED framework to the scientific community and illustrate that, with appropriate supporting data, questions such as these could be explored via both in-silico and experimental trials.

## Conclusion

A novel framework for the pre-clinical and clinical treatment planning of high Z NP-enhanced photon radiotherapy was developed and its applicability tested in silico for the medical exemplar of neoadjuvant (preoperative) breast cancer MV photon radiotherapy. Whilst a definite conclusion about the viability of the selected high Z NP radiosensitisation agents for the selected photon radiotherapy medical exemplar could not be obtained, it was illustrated with the RBED framework that the extent of possible biological response was great than expected via the dose deposition alone. Further exploration of the role of high Z NP radiosensitisation agents in photon radiotherapy is warranted and it is anticipated that this framework will assist the scientific community in future high Z NP-enhanced in-silico, pre-clinical and clinical trials.

## Abbreviations

AuNP: gold nanoparticle
DEFM: dose enhancement figures of merit
NP: nanoparticle
LEM: local effect model
RBE: Relative Biological Effectiveness
RBED: Relative Biological Effective Dose
